# Huntington’s Disease: Understanding Its Novel Drugs and Treatments

**DOI:** 10.7759/cureus.47526

**Published:** 2023-10-23

**Authors:** Hitaansh Dhingra, Shilpa A Gaidhane

**Affiliations:** 1 Medicine, Jawaharlal Nehru Medical College, Datta Meghe Institute of Higher Education and Research, Wardha, IND

**Keywords:** selective serotonin reuptake inhibitor, cag repeat, pluripotent stem cells, molecular pharmacology, huntington disease

## Abstract

An inherited neurodegenerative ailment called Huntington's disease (HD) of gradual physical impairment, cognitive decline, and psychiatric symptoms. It is brought on by a mutation of the HTT gene, which causes aberrant huntingtin protein buildup in neurons. This predominantly affects the striatum and cerebral cortex, where neuronal malfunction and eventual cell death follow. The quality index of life for both patients and their families is significantly impacted when symptoms first appear in mid-adulthood. An overview of the available therapies for HD is given in this article. Although HD has no known treatment options, there are several that try to lessen symptoms and reduce the disease's development. By lowering involuntary movements, pharmaceutical treatments like tetrabenazine and deutetrabenazine focus on motor symptoms. Antidepressants and antipsychotic medicines are also used to manage the mental and cognitive symptoms of HD. The investigation of prospective gene-based medicines is a result of research into disease-modifying medications. Reduced synthesis of mutant huntingtin protein is the goal of RNA interference (RNAi) strategies, which may halt the course of illness. Additionally, continuing research into Clustered Regularly Interspaced Short Palindromic Repeats and CRISPR-associated protein 9 (CRISPR-Cas9) and other gene editing methods shows promise for reversing the genetic mutation that causes HD. Individuals with HD can benefit from non-pharmacological therapies such as physical therapy, speech therapy, and occupational therapy to increase their functional abilities and general well-being. Supportive treatment, psychiatric therapy, and caregiver support groups are also essential in addressing the difficult problems the illness presents. In conclusion, tremendous progress is being made in the domain of HD treatment, with an emphasis on symptom control, disease modification, and prospective gene-based therapeutics. Even though there has been significant improvement, more study is still required to provide better therapies and ultimately discover a solution for this debilitating condition.

## Introduction and background

Huntington's disease (HD) is primarily caused by the enlargement of the trinucleotide repeat (CAG) inside the huntingtin gene (HTT) located on chromosome 4. This genetic alteration is responsible for the development of HD, a neurological disorder that exhibits complete penetrance and follows an autosomal dominant inheritance pattern. The sickness is characterized by a combination of motor symptoms, progressive cognitive decline, and mental disorders. The observed clinical symptoms may be attributed to neuronal dysfunction, which originated in the medium spiny neurons of the striatum and then spread to the cortex and other regions of the brain. Currently, there are no officially approved disease-modifying medications for HD. However, there is promising research being conducted on innovative approaches that target the genetic origins of this disorder. There is an urgent need for the development of enhanced symptomatic therapies since the current absence of curative treatments and the presence of disruptive neuropsychiatric symptoms need such advancements [1-3].

HD is a neurodegenerative condition characterized by a pathological amplification of CAG repeats within the HTT gene, resulting in an extended polyglutamine tract. A negative correlation has been shown between the length of the CAG repeat and the age at which symptoms first appear. The manifestation of symptoms typically occurs at a mean age of 45 years [4].

Additionally, the salient clinical features of HD are such that the individual may exhibit a range of symptoms associated with motor and cognitive dysfunction. These symptoms include agitation, irritability, apathy, anxiety, hallucinations, inconsistent eye movements, depression, dysfunctional olfaction, uncontrollable movements, balance and gait disturbances, chorea, jerky movements, staggering, swaying, and an irregular gait resembling drunkenness. Difficulties may also arise in tasks requiring physical dexterity, such as slow voluntary movements, trouble initiating actions, and an inability to regulate the power and pace of movements. Additionally, the individual may experience a delay in response time, overall weakness, weight loss, speech impediments, stubbornness, rigidity, bradykinesia (difficulty initiating and maintaining movements), and, less frequently, extreme chorea. These symptoms can lead to a significant decrease in weight, loss of ability to walk, inability to speak, dysphagia, increased risk of choking, and an inability to perform activities of daily living independently.

## Review

Methodology

Literature Search

We utilized various databases such as PubMed and Google Scholar. Our search included specific keywords relevant to our study, including "Huntington’s disease", "treatment", "newer drugs", "epigenetics" and "pharmacotherapy". We also manually searched the reference lists of relevant articles to identify additional studies.

Inclusion and Exclusion Criteria

Incorporated in our research were studies that explored the various aspects of HD, such as pathology, clinical features, treatment, and symptomatic relief. However, we opted to exclude non-English studies and those that were paid to access.

Data Extraction and Synthesis

In order to conduct our analysis, we obtained information from each study that was included in the research. To evaluate this collected information effectively, we employed a narrative approach, which allowed us to provide an overview while highlighting important and current details.

Data Analysis

**Figure 1:**
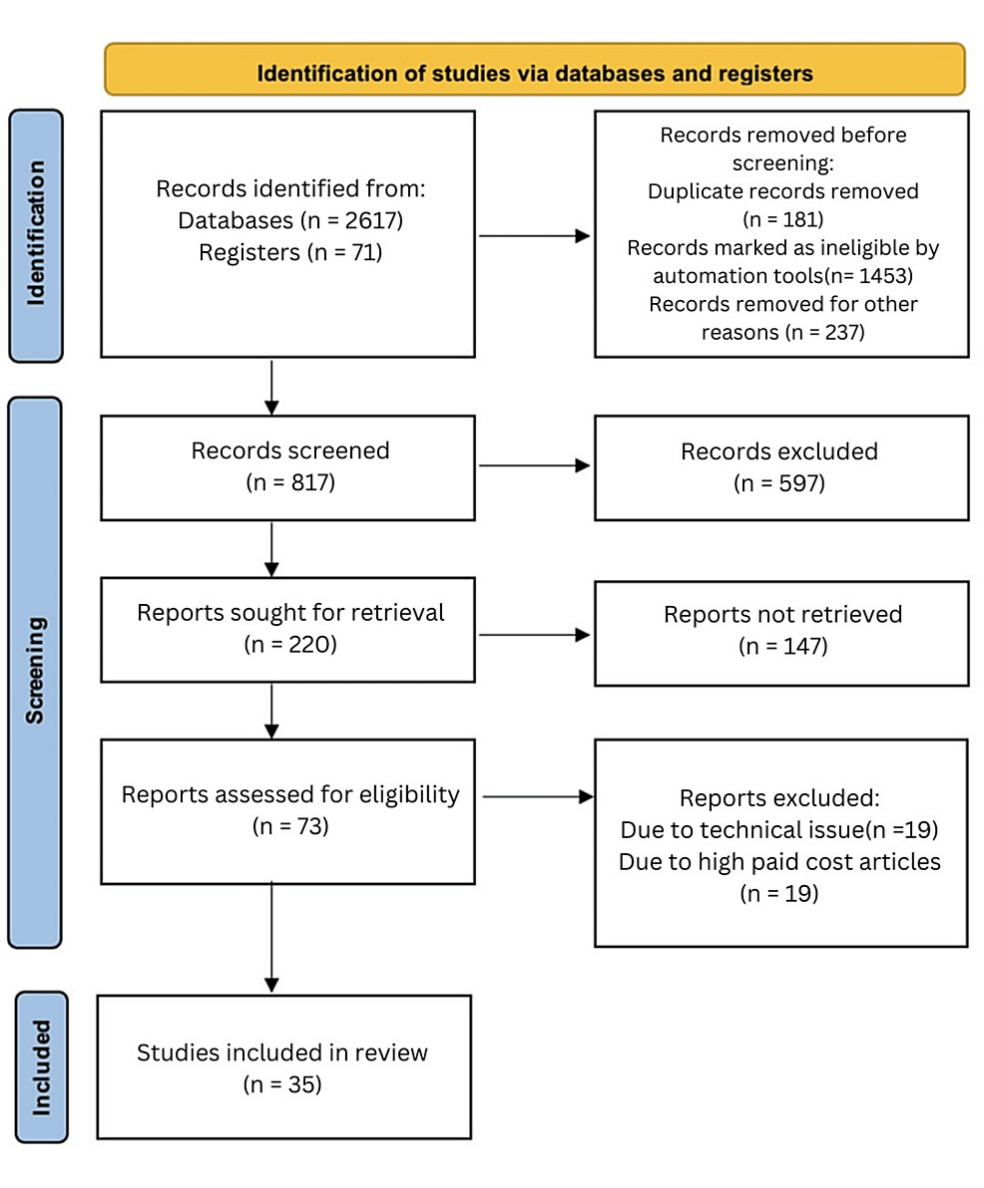
Search strategy using the PRISMA method PRISMA: Preferred Reporting Items for Systematic Reviews and Meta-Analyses

In our analysis, we used a qualitative method to study the data. We focused on identifying repeating themes and patterns that emerged from the studies included in our research. Figure 1 shows the search strategy utilized.

Given the current state of clinical research, there is a lack of effective identification of disease-modifying drugs for HD. As a result, the prevailing therapeutic approaches mostly focus on managing symptoms rather than addressing the underlying cause of the condition. The present treatment strategies prioritize the effective management of physical, cognitive, and mental symptoms, aiming to improve the overall quality of life for those impacted by this particular ailment. This section provides an overview of the existing therapeutic approaches used for the treatment of this particular neurodegenerative disorder [5-10].

Currently, there is a lack of commercially available pharmaceutical interventions that have been specifically developed for the purpose of influencing the progression of HD. The treatment modality only focuses on alleviating symptoms. Tetrabenazine is the only medicine approved for the treatment of choreiform movements associated with HD. Clinical trials evaluating the efficacy of cholinesterase inhibitors, a class of drugs often prescribed for managing cognitive impairments in individuals with Alzheimer's disease, have largely shown unfavourable results in the context of HD. Pharmacological interventions often performed in non-HD patients are often applied to address the uncomfortable symptoms associated with mental illness. For example, the administration of atypical antipsychotics is often used in the management of psychosis, while the treatment of depression often involves the utilization of antidepressants such as selective serotonin reuptake inhibitors (SSRI) or selective norepinephrine reuptake inhibitors (SNRI). The therapeutic treatments discussed mostly rely on case studies or small series, with the exception of a solitary study that investigates the utilization of venlafaxine. Various paramedical professions, such as speech and language therapy, rehabilitation, nursing, and social aid, are used in the care and treatment of individuals who have been diagnosed with HD [11-14].

Caspase inhibition/anti-apoptotic agents

The tetracycline antibiotic minocycline is a caspase inhibitor, but it also has antioxidant and cytokine-modulating characteristics, like many of the substances discussed here [15,16].

Transglutaminase inhibition

Transglutaminase is responsible for the cross-linking of glutamine residues within the huntingtin protein. Promising outcomes have been seen with the use of transglutaminase inhibitors, such as cystamine [17].

Enhanced delivery methods

**Figure 2:**
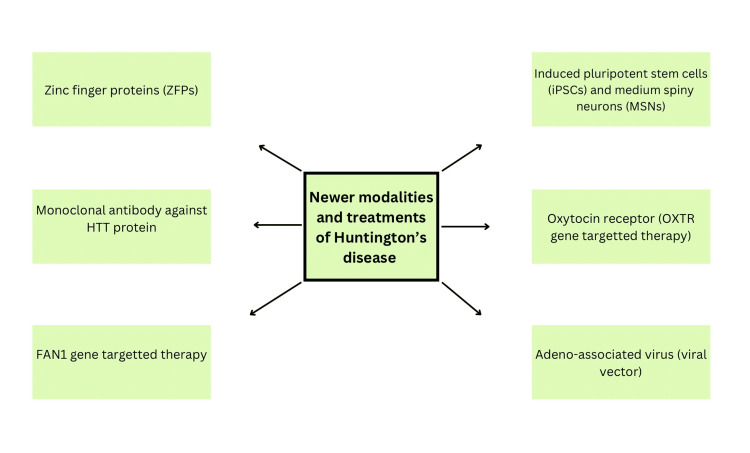
Newer modalities and treatments of Huntington’s disease The figure is the author’s own creation.

For several days or weeks, various experiments included the continuous infusion of radiolabeled small interfering RNA (siRNA), which was not encoded in a viral vector, into the putamen of monkeys via convection-enhanced administration. This administration method led to the diffusion of siRNA and subsequent lowering of HTT across the whole striatum. Additional non-viral approaches are being studied as potential strategies for the improvement of the dispersion of RNA interference (RNAi) chemicals and their delivery inside the nervous system (CNS). In the future, it will be likely that exosomes or other strategies resembling "Trojan horses" might enable the administration of intravenous dosages. This is due to the ability of single-stranded RNA molecules (ssRNA) to easily traverse the brain parenchyma and enter cells [3,18,19,20]. Figure 2 depicts the newer modalities and treatments of HD.

Zinc fingers

These structural motifs are artificially generated yet have natural origins. They have the ability to bind certain DNA sequences and are used as therapeutic agents that target DNA. Zinc finger proteins (ZFPs) used for therapeutic purposes are often engineered with a zinc finger array that is customized for matching the specific DNA sequence of use. Each zinc finger within the array corresponds to a set of three DNA bases and is fused to a functional domain that facilitates the desired biological activity. Examples include the DNA-cleaving zinc finger nucleases (ZFNs) and the gene-expression-regulating zinc finger transcription factors (ZFTRs) [21].

ZFNs have the potential to do targeted "genome editing", however at this time, the technique is not accurate or predictable enough to be used therapeutically in post-mitotic patient neurons. Zinc fingers, however, are trustworthy enough to consider the research and development of a therapeutic ZFTR. The zinc finger array is required to specifically interact with a nucleotide sequence in proximity to the 3' terminus of the DNA sense strand. This interaction is crucial for the precise positioning of the transcriptional repressor in close proximity to the HTT promoter region. Due to these constraints, it is challenging to prevent unintended binding to other genes [3].

Induced pluripotent stem cells

They provide an additional alternative for cell replacement treatment in the context of HD. Due to their origin from the patient, induced pluripotent stem cells (iPSCs) have the advantage of facilitating autologous transplantation, hence, mitigating the risk of immunological rejection and obviating the need for post-transplant immunosuppression treatment. In a particular research investigation, the striatum of mice was subjected to transplantation of induced neural stem cells (iNSCs) derived from fibroblasts. These transplanted cells successfully differentiated into neurons that displayed markers characteristic of medium spiny neurons (MSNs). The cells that were implanted also exhibited an extended length of survival exceeding six months. Nevertheless, the application of these donor cells in cell therapy is limited as a result of HD being an inherited disorder. Consequently, prior to using autologous cell therapy, the genetic modification of the HD mutation emerges as a crucial aim. In a particular investigation, the HD mutation present in iPSCs was rectified prior to the transplantation of these cells into mouse models with HD. Although there was no observed alteration in motor skills, the transplanted cells successfully differentiated into neurons and exhibited the expression of markers specific to MSNs [22,23].

Epigenetics

HD is a hereditary neurodegenerative condition that has a significant influence on both motor and cognitive abilities by affecting the MSNs in the striatum. The mutHTT protein induces neuronal toxicity via the amplification of the PolyQ pathway. Therapeutic options are now being explored in many preclinical models and clinical investigations. The aforementioned therapy interventions have been linked to a decrease in the quantities of mutHTT seen in the genome, mRNA, or degradable proteins. In addition to therapeutic treatments, the process of modifying post-translational mutHTT is also a biological phenomenon. The possible use of stem cell treatment in conjunction with conventional medicines may be contemplated as a reaction to the degeneration of striatal neurons. The conducted clinical research has the capacity to identify essential biomarkers that might be used in the treatment of HD. The advent of innovative gene editing techniques has allowed the thorough investigation and modelling of human diseases at the cellular and molecular levels. The approaches of ZFN, TALEN, and CRISPR/Cas9 have revealed several changes. These systems have a role in controlling the transcriptional activity of genes in various biological environments, such as iPSCs and genetic sequences. The previously indicated techniques, which are used to create cellular populations with varying CAG repeat counts, may also be utilized to correct the mutations that give rise to HD. In recent years, there has been a notable increase in the volume of scientific investigations focused on the replication of molecular pathways and genetic disorders. TALEN and ZFN are seminal instances of synthetic nucleases. In comparison to Clustered Regularly Interspaced Short Palindromic Repeats (CRISPR) systems, these particular systems demonstrate a greater level of simplicity.

Researchers use human iPSCs to produce cell populations that are linked to specific disease states, with the aim of exploring the molecular pathways behind human disorders. The use of iPSCs derived from the patient offers a potential avenue for exploring the cellular mechanisms underlying dementia in an in vitro setting. iPSCs have the capacity to undergo differentiation into specific subtypes of neurons, hence, facilitating the investigation of specific neuronal populations that are pertinent to the pathogenesis of dementia. iPSCs with distinct characteristics have been used in the simulation of many neurodegenerative disorders, such as Alzheimer's disease and HD. The use of iPSC technology in therapeutic contexts is accompanied by a multitude of obstacles. The combination of iPSCs with genome editing technology holds promise for the development of innovative pharmaceuticals and therapies targeting neurodegenerative illnesses in the human populace [24,25].

Venlafaxine, desvenlafaxine and duloxetine

These can be administered when an SNRI is chosen. Clozapine should be suggested as the choice of treatment for HD patients who have significant Parkinsonian symptoms but are akinetic in nature. When the patient exhibits persistent ideation resembling psychotic symptoms, it may be advantageous to consider a therapeutic approach using serotonergic antidepressants in conjunction with an atypical neuroleptic [26].

By means of the isoenzymes CYP1A2, CYP2D6, etc. in the liver, mirtazapine is extensively metabolized [26]. There are potential advantages for those who suffer from depression and concurrently exhibit symptoms of anxiety and sleep disturbances [27].

Oxytocin receptor (OXTR)

The gene responsible for encoding the oxytocin receptor, known as OXTR, has variations that have been linked to apathy and reduced social cognition. These variations may serve as markers for hypothalamic degeneration in HD, leading to impaired oxytocin function and its association with neuropsychiatric symptoms [28]. Polymorphisms in the OXTR have the potential to have an impact on the optimal development of social and cognitive capacities [26].

Vesicular monoamine transporter type 2 (VMAT2) inhibitors

Except for persons who experience depression, vesicular monoamine transporter type 2 (VMAT2) inhibitors are generally regarded as the most effective pharmacological interventions for the treatment of chorea. This is due to an indication of it worsening depressive episodes and increasing the likelihood of suicidal thoughts [29].

Antipsychotics

They are used as a treatment for chorea in cases where VMAT2 inhibitors are contraindicated, such as in individuals with depression, or where VMAT2 inhibitors prove ineffective in managing symptoms. Additionally, they have the potential to assist in managing mental symptoms associated with HD. The preference for second-generation antipsychotics over first-generation drugs is mostly attributed to their reduced propensity for extrapyramidal side effects. They work by inhibiting serotonin receptors, particularly 5-HT2A and D2 dopamine receptors [26].

First-generation antipsychotics are only used in instances of severe chorea that are unresponsive to VMAT inhibitors and second-generation antipsychotics, owing to their heightened efficacy [26].

Other drugs

Long-term administration of benzodiazepines, including clonazepam and lorazepam, is not recommended; however, they may be used on a temporary basis to alleviate intense bouts of chorea [26].

According to recent research, the use of flavonoids has been proposed due to their potential to mitigate cellular stress and exhibit anti-inflammatory and anti-apoptotic properties on cellular systems [30].

Tetrabenazine (TBZ)

A dopamine uptake inhibitor that limits vesicle formation has been demonstrated to have anti-chorea effects amongst patients and was the first to receive FDA approval [29].

Monoclonal antibody against HTT protein

An alternative investigation has proposed that the injection of a monoclonal antibody, which selectively targets the HTT protein, might potentially decrease the protein's concentration inside the cell. This finding serves as evidence for the capacity of monoclonal antibodies to impede the pathological mechanisms involved in the in vivo propagation of mutant huntingtin (mHTT) [31].

To introduce these agents into the organism, several research studies have used viral vectors such as the adeno-associated virus (AAV). These vectors encapsulate the RNA molecules and possess genomes that are linked with enhancers and promoters [32].

Due to its inherent instability, repeated DNA sequences have a tendency to undergo amplification in the HTT gene, namely in the form of an expanded CAG repeat. This phenomenon, referred to as somatic instability, leads to an accumulation of additional repeats during the lifespan of individuals affected with HD. The rate of expansion exhibits variability across different tissues and cell types. Notably, neurons have a higher rate of expansion compared to glia. This phenomenon is particularly evident in striatal and cortical cells, which are known to be more prone to degeneration in HD [33].

FAN1 gene targetted therapy

Recent studies done on a large cohort of individuals with HD have shown that DNA repair mechanisms play a pivotal role in shaping the progression of this disorder. Furthermore, it has been observed that the genes implicated in HD also have an impact on the development and manifestation of other trinucleotide repeat illnesses. The FAN1 gene, which encodes a nuclease responsible for cleaving DNA during the repair process of crosslinks between DNA strands, had a significant influence as a modifier gene. FAN1 has been identified as a potential protective factor in HD, perhaps exerting its effects via efficient repair of loopouts that arise at CAG repeats [34].

Pridopidine

**Figure 3:**
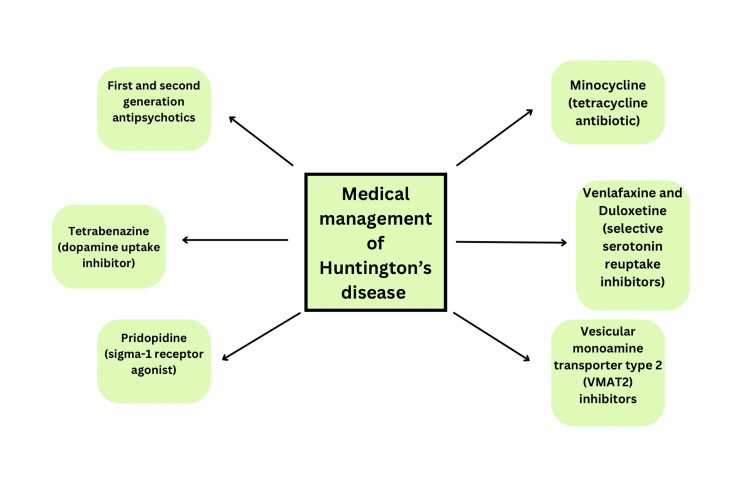
Medical management of Huntington’s disease The figure is the author’s own creation.

Pridopidine was formerly referred to as ACR16 is an example of a sigma-1 receptor agonist. It exhibited the ability to reinstate homeostatic synaptic plasticity and rectify calcium signalling abnormalities in neurons affected by HD. Additionally, it demonstrated the capacity to prevent cell death induced by mHTT in HD mouse neurons and human iPSCs derived from HD patients. Notably, the efficacy of pridopidine was negated in neurons lacking the sigma-1 receptor, as well as in lymphoblasts obtained from HD patients and human iPSCs derived from individuals with HD [34,35]. Figure 3 depicts the medical management of HD.

## Conclusions

HD is still a hard-to-treat condition that requires cutting-edge, efficient treatments. Pharmacogenetic research's long-term objective is to utilize genotype information to forecast how well a certain medicine will treat a patient and so reduce the risk of any unfavourable side effects from being administered. In order to address the challenges posed by movement disorders, depression, anxiety, and psychosis, which have a substantial negative impact on the overall well-being of patients, the use of pharmacogenetic data may be employed to enhance the effectiveness of HD anti-choreic, antidepressant, and antipsychotic medications.
